# Supplementary material for: COZOID: contact zone identifier for visual analysis of protein-protein interactions
Source: BMC Bioinformatics. 2018 Apr 6;19:125. doi: 10.1186/s12859-018-2113-6 (PMC5889581; doi:10.1186/s12859-018-2113-6)
Supplement: Supplementary file 2 — Software build. Executable binary file of the software tool. (ZIP 118,784 kb) [file 12859_2018_2113_MOESM2_ESM.zip › cozoid/platform/docs/testng-6.5.1-javadoc.zip!/deprecated-list.html]

Deprecated List (TestNG)


---


|  |  |  |
| --- | --- | --- |
| **Overview** \| Package \| Class \| **Tree** \| **Deprecated** \| **Index** \| **Help** | |  |
| PREV   NEXT | **FRAMES**    **NO FRAMES**     **All Classes** |  |


---


## **Deprecated API**


---

**Contents**

- Deprecated Annotation Types- Deprecated Methods- Deprecated Annotation Type Elements

**Deprecated Annotation Types**

org.testng.annotations.Configuration
  
          *Use @BeforeSuite, @AfterSuite, @BeforeTest, @AfterTest,
\@BeforeGroups, @AfterGroups, @BeforeClass, @AfterClass, @BeforeMethod,
\@AfterMethod*

org.testng.annotations.ExpectedExceptions
  
          *Use @Test(expectedExceptions = "...")*

**Deprecated Methods**

org.testng.TestNG.configure(Map)
  
          *use new configure(CommandLineArgs) method*

org.testng.TestNG.getDefault()
  
          *since 5.1*

org.testng.IMethodInstance.getInstances()
  
          *Use getInstance()*

org.testng.ITestNGMethod.getInstances()
  
          *Use getInstance().*

org.testng.ISuite.getInvokedMethods()
  
          *Use getAllInvokedMthods().*

org.testng.ITestNGMethod.getMethod()
  
          *This method is deprecated and can now return null. Use
getConstructorOrMethod() instead.*

org.testng.annotations.IParameterizable.getParameters()
  
          *Use @Parameters*

org.testng.TestNG.isJdk14()

org.testng.xml.XmlTest.setClassNames(List)
  
          *use setXmlClasses*

org.testng.TestNG.setHasFailure(boolean)
  
          *since 5.1*

org.testng.TestNG.setHasFailureWithinSuccessPercentage(boolean)
  
          *since 5.1*

org.testng.TestNG.setHasSkip(boolean)
  
          *since 5.1*

org.testng.TestNGAntTask.setListener(String)
  
          *Use "listeners"*

org.testng.TestNG.setTestNGVersion()
  
          *The TestNG version is now established at load time. This
method is not required anymore and is now a no-op.*

**Deprecated Annotation Type Elements**

org.testng.annotations.Configuration.parameters
  
          *Use @Parameters*

org.testng.annotations.Factory.parameters
  
          *Use @Parameters*

org.testng.annotations.Test.parameters
  
          *Use @Parameters*

org.testng.annotations.Test.sequential
  
          *Use singleThreaded*

---


|  |  |  |
| --- | --- | --- |
| **Overview** \| Package \| Class \| **Tree** \| **Deprecated** \| **Index** \| **Help** | |  |
| PREV   NEXT | **FRAMES**    **NO FRAMES**     **All Classes** |  |


---
